# *In vivo* calcium imaging from dentate granule cells with wide-field fluorescence microscopy

**DOI:** 10.1371/journal.pone.0180452

**Published:** 2017-07-12

**Authors:** Yuichiro Hayashi, Satoshi Yawata, Kazuo Funabiki, Takatoshi Hikida

**Affiliations:** 1 Osaka Bioscience Institute, Suita, Osaka, Japan; 2 Medical Innovation Center, Kyoto University Graduate School of Medicine, Kyoto, Japan; 3 Frontier Research Center for Post-genome Science and Technology, Hokkaido University, Sapporo, Japan; Pennsylvania State Hershey College of Medicine, UNITED STATES

## Abstract

A combination of genetically-encoded calcium indicators and micro-optics has enabled monitoring of large-scale dynamics of neuronal activity from behaving animals. In these studies, wide-field microscopy is often used to visualize neural activity. However, this method lacks optical sectioning capability, and therefore its axial resolution is generally poor. At present, it is unclear whether wide-field microscopy can visualize activity of densely packed small neurons at cellular resolution. To examine the applicability of wide-field microscopy for small-sized neurons, we recorded calcium activity of dentate granule cells having a small soma diameter of approximately 10 micrometers. Using a combination of high numerical aperture (0.8) objective lens and independent component analysis-based image segmentation technique, activity of putative single granule cell activity was separated from wide-field calcium imaging data. The result encourages wider application of wide-field microscopy in *in vivo* neurophysiology.

## Introduction

To record neural activity in living animals, extracellular electrophysiological recording has been widely used [1–3]. This technique offers high temporal resolution and low invasion of brain tissue, but recording the same neurons for a long period of time (days to weeks) and to determine their precise location and cell types are difficult. In contrast, calcium imaging with genetically-encoded calcium indicators provides a long-term, cell-type-specific method of recording neural activity [4,5]. Because two-photon microscopy provides deep tissue penetration and optical sectioning capability, it is suitable for *in vivo* calcium imaging of deep brain structures. However, because each pixel in an image is sampled serially in two-photon microscopy, an inevitable tradeoff exists between the size of imaging area and frame rate. Two-photon microscope using wide-field excitation or multi-beam scanning technique offers wide field of view at high frame rate [6,7]. However, their applicability to *in vivo* calcium imaging is yet to be demonstrated. On the other hand, wide-field microscopy have also been used for visualizing neural dynamics in various brain structures [8–12]. Unlike conventional two-photon microscopy, all pixels in an image are sampled simultaneously in wide-field microscopy. Therefore, this method is advantageous over two-photon microscopy for high speed, high resolution imaging. However, due to the lack of optical sectioning capability, axial resolution of wide-field microscopy is worse than that of confocal or two-photon microscopy. At present, it is unclear whether wide-field microscopy is applicable to densely packed small cells. To examine the applicability of wide-field fluorescence microscopy for smaller sized neurons, we recorded calcium activity of dentate granule cells (GCs) having a small soma diameter of approximately 10 micrometers. By employing high-numerical aperture (NA) (0.8) objective lens and independent component analysis (ICA)-based cell sorting technique, activity of individual hippocampal GCs in head-restrained mouse were separated.

## Materials and methods

### Optical path

**Fig 1:**
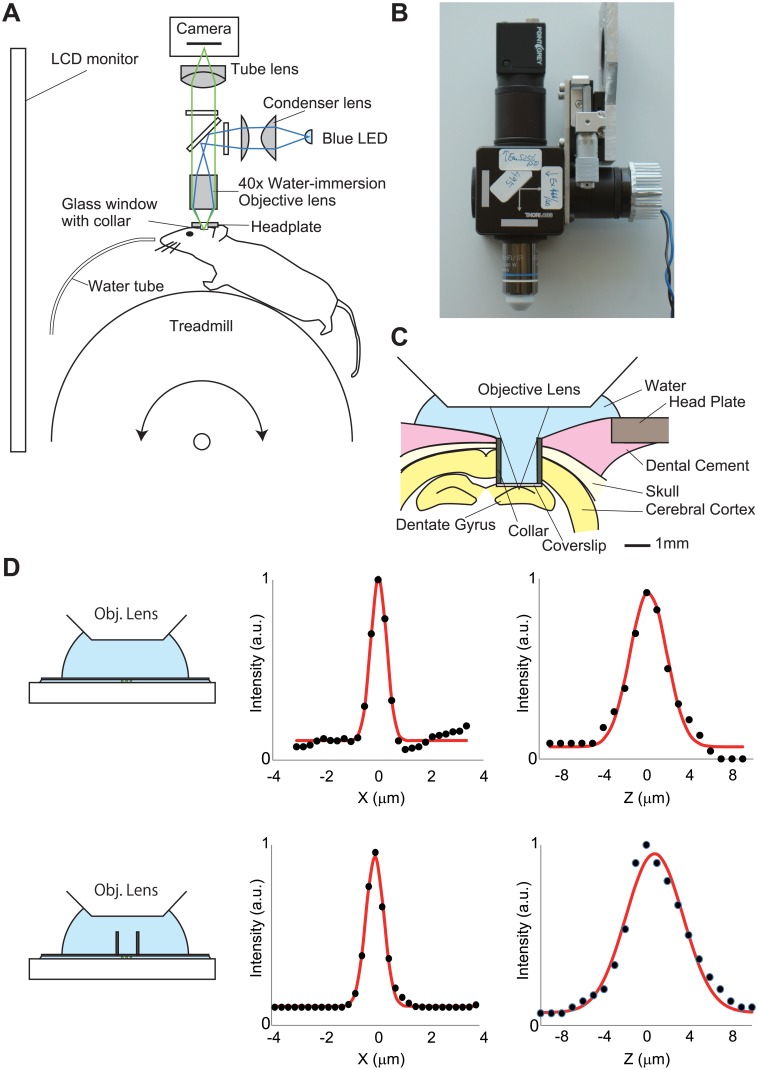
Wide-field imaging system for an awake head-restrained mouse. (A) Simplified schematic of the endoscope imaging system. The blue line indicates the illumination pathway and the green line indicates the light collection pathway. Illumination light from a blue LED was collected with a condenser lens, passes through an excitation filter, reflects off a dichroic mirror, and irradiated through an objective lens. The fluorescence image was focused on a CMOS camera. (B) Image of the endoscope system. (C) Schematic of experimental setup showing the chronic window implant above the dentate gyrus. (D) Point spread function of the imaging setup with (lower) and without (upper) the cannula. Lateral (center) and axial (right) intensity line profiles across a 0.5 μm fluorescent bead (black dots) were shown. The read lines indicate Gaussian fits.

A simplified schematic of the optical path is shown in Fig 1A. Excitation light was emitted from a blue LED (LXK2-PB14-P00; Lumileds, Aachen, Germany). The light passed through an excitation filter (FF480/40-25; Semrock, Rochester, NY) and then reflected by a dichroic mirror (FF506-Di02-25x36; Semrock) onto the tissue through a water-immersion objective lens (40×0.8NA; Olympus, Tokyo, Japan). Fluorescent emissions collected by the objective lens were passed through the dichroic mirror and an emission filter (FF535/50-25; Semrock). The fluorescence image, focused by a tube lens (Nikkor 50mm f/1.8D, Nikon, Tokyo, Japan), was captured by a CMOS camera (FL3-U3-13S2M-CS, FLIR Systems, Willsonville, OR). Detailed construction of the overhead fluorescence microscope unit is shown in S1 Fig.

### Resolution measurements

To determine resolution values, lateral and axial intensity profiles of the 0.5 μm fluorescent bead (C14837, Thermo Fischer Scientific, Waltham, MA) images were fit to Gaussian curves. We determined the full-width-at-half-maximum (FWHM) of each curve fit.

### Animals and surgery

All animal care and use was in accordance with the protocols approved by Hokkaido University institutional animal care and use committee (project license number: 16–0042). Adult male C57BL/6J mice (10–16 weeks of age) were anesthetized with isoflurane, and then injected with 0.1mg/kg buprenorphine. The skull was exposed, and a small hole (<0.5 mm) was made over the right hemisphere (1.5 mm lateral to the midline, 2.3 mm posterior to the bregma). AAV1-syn-GCaMP6f-WPRE (University of Pennsylvania vector core) was diluted to 5×10^12^ particles/mL in phosphate-buffered saline (PBS), and 150 nL was injected into granular layer of the dentate gyrus (1.6 mm ventral from the brain surface). One week after the viral injection, the animal was anesthetized and a 1.9 mm diameter craniotomy was performed. The dura was removed, and the underlying cortex and CA1 was aspirated. A stainless-steel cannula (1.81 mm outer diameter, 1.45 mm inner diameter, 1.8 mm height) covered by a cover glass (0.12 mm thickness) was inserted over the dorsal dentate gyrus. A titanium head plate (25×10 mm, 1mm thickness) and the skull were glued with dental cement (Shofu, Kyoto, Japan).

### VR system

**Fig 2:**
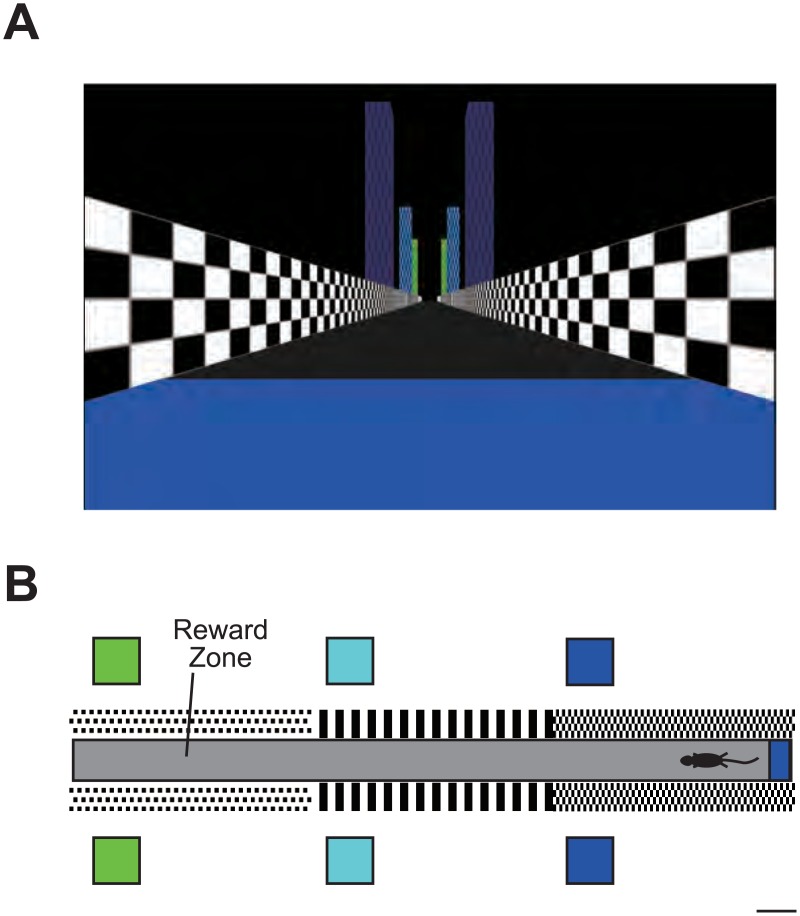
VR system for mice. (A) View from start point of the virtual linear track. (B) Top view of the track. Scale bar, 10 cm.

A 24 inch LCD monitor (2407WFP, Dell, Round Rock, TX) placed ~26 cm from the mouse’s head, and the VR environment image displayed on the monitor covered 90° and 77° of the mouse’s horizontal and vertical field of view, respectively (Fig 2A). Mice were head-restrained with their limbs resting on a freely rotating treadmill consisting of a 20 cm diameter Styrofoam ball with a metal shaft. Ball rotations associated with mouse locomotion were read at 60 Hz with a computer mouse (G300, Logitech, Lausanne, Switzerland) placed under the ball. The length of the virtual track was 1.8 m. Mice received a small sugar water reward in an unmarked reward zone (1.5 m from the start point of the track) (Fig 2B). Once the mice reached the end of the track, they were teleported back to the start of the track. The Blender Game Engine (www.blender.org) and its Python interface were used to generate the VR environment.

### Behavioral training

At least 2 week after the cranial window implantation, the mice underwent water restriction (2 mL per day), and training in the virtual linear track (two 5 min session per day) began. After 3–4 weeks of training, mice achieving a high performance levels (8 rewards per session) were used for recording session.

### Imaging session

Excitation light intensity was approximately 0.04 mW/mm^2^. Images were captured using FlyCapture2 software (FLIR Systems, Willsonville, OR) at 10 Hz for 5 minutes.

### Image processing

We used ImageJ1.49q (National Institute of Health) and MATLAB 2013a (Mathworks, Natick, MA) for all analyses. We first applied a spatial down-sampling of 4× to the image cropped to the area containing the putative neural activity. Then we corrected lateral displacements of the brain by image translation using TurboReg [13]. We used an averaged image of the entire frames as a reference for image alignment. Spatial filters corresponding to individual cells were identified using a PCA and ICA-based algorithm (Cellsort 1.0) (Mukamel et al., 2009).

### Calcium activity data analysis

Ca^2+^ activity was extracted by applying the spatial filters to the time-lapse image. The Ca^2+^ transients were identified by searching each trace for local maxima that had a peak amplitude of more than 3× standard deviation from the baseline (defined as the average of the trace calculated across the entire sessions).

## Results

### Wide-field fluorescence imaging of dentate GCs in awake behaving mice

To test the feasibility of wide-field microscopy for *in vivo* calcium imaging from small, densely packed neurons, we record activity from GCs of the dentate gyrus. These cells have small soma (approximately 10 μm) and are tightly packed in the granular layer [14], therefore, imaging optics should have high lateral and axial resolution. In *in vivo* calcium imaging with wide field fluorescence microscopy, gradient refractive index (GRIN) lens is often used as objective lens. However, because of its low NA (<0.6) and significant optical aberrations, the optical resolution of singlet GRIN lens is not as good as conventional microscope objectives. To achieve high axial resolution, we used a 40×, 0.8NA water-immersion objective lens (Fig 1A and 1B). The lateral and axial full-width at half-maximum (FWHM) of the point spread function was 0.70 μm and 4.03 μm (Fig 1D, upper). For *in vivo* imaging from dentate gyrus, we used glass window with stainless-steel cannula (Fig 1C). Because the cannula blocks light rays of the object, effective numerical aperture is reduced to ~0.5, which impairs the spatial resolution. Therefore, we also measured PSF in the presence of the cannula. The lateral and axial FWHM are 0.81μm and 6.36 μm, respectively, which are less than soma size of the GCs (Fig 1D, lower). To test whether this imaging setup can resolve individual GCs, three-dimensional image of formaldehyde-fixed brain tissue expressing GCaMP6f [15] at 2 μm step, and multiple XZ views were shown (S2 Fig). In each XZ plane contains one or more GCaMP6f-labeled neurons, and the shape of individual neurons were clearly visualized (S2 Fig). These results suggest that the spatial resolution of this imaging setup is sufficient for cellular-level calcium imaging of dentate GCs. However, unlike confocal or two-photon microscopy, fluorescence from out-of-focus plane is also received by the image sensor in wide-field microscopy. Therefore, each pixel in the image obtained by this method can contain a mixture of signals from multiple cells [16]. This mixed signal can be separated into signal from individual cells using statistical techniques [17–20]. We used principal and independent component analysis (PCA and ICA)-based method (Cellsort)[17] for cell sorting.

The mouse was head-fixed on a spherical treadmill that allows the mouse to run freely (Fig 1A) [21]. A custom virtual-reality (VR) system for rodents [22–25] was used to present spatial cues. A liquid crystal display (LCD) monitor installed in front of the mouse displayed a computer-generated image of a virtual linear track (Fig 2).

Mice were injected with an adeno-associated virus (AAV) expressing GCaMP6f into the granular layer of the dentate gyrus, and then implanted with a glass window. After 2–4 week of recovery, mice were trained to obtain a sugar water reward (10% sucrose, 4 μL) at a hidden goal zone in the virtual linear track.

### Activity profile of the dentate GCs

**Fig 3:**
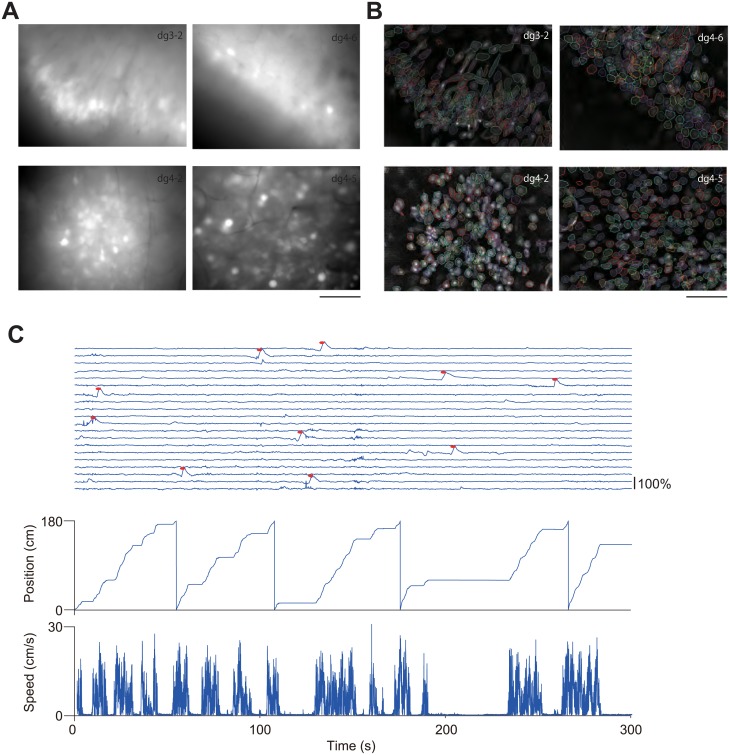
Imaging the dentate gyrus in behaving mice. (A) Averaged fluorescence images across entire recording session. Scale bar, 100 μm. (B) Contours of putative cell bodies identified with a PCA and ICA-based cell sorting algorithm (Cellsort 1.0, Mukamel et al., 2009). Scale bar, 100 μm. (C) Traces of dF/F calcium signal were shown. Identified calcium transients are shown as red dots. The position of the mouse along the virtual track is shown at the bottom. Animal position and speed in the virtual track was shown in the bottom.

We recorded fluorescent images from the dentate gyrus at 10 Hz for 5 min while mice were performing the VR task (running speed: 2.2 ± 0.96 cm/s, reward rate: 10.0 ± 4.8 reward/session, mean ± SEM, n = 4)(Fig 3A, S1 File). We then extracted fluorescent signals of individual cells from the image data using Cellsort program (Mukamel et al., 2009). The shape and size of spatial filters derived by ICA suggested that the method successfully identified individual cells (Fig 3B). We then applied these spatial filters to extract individual calcium activity profiles, and detected calcium transients from the extracted waveform (Fig 3C). Of 967 cells (n = 4 mice) detected by ICA, calcium activity of the GCs was very sparse (0.17 ± 0.0057 events/min, mean ± SEM), which is consistent with previous *in vivo* calcium imaging studies using two-photon microscopy [26,27].

## Discussion

Wide-field microscopy have been used to image calcium activity in various brain regions from awake behaving animals [10–12]. Noteworthy, owing to its simple mechanism, the method can be miniaturized for imaging from free-moving rodents, which have expanded the scope of applications [12]. A major disadvantage of wide-field microscopy is lacking of optical sectioning capability, and hence poor axial resolution. To separate individual activity of small, densely packed cells, we first used high-NA (0.8) objective lens. This lens yielded high lateral and axial resolution, which is enough to separate individual dentate GCs (Fig 1D and S2 Fig). Second, we used PCA-ICA based cell sorting software (Cellsort)[17] (Fig 3B) to separate overlapping fluorescence from adjacent cells. Spatial filters derived from the software appear to represent individual GCs with minimal overlap (Fig 3B), suggesting that the software successfully extracted individual cell activity. Activity traces of the dentate GCs during rest and running on the treadmill were highly sparse (Fig 3C), consistent with previous electrophysiological studies [26,28–31] and calcium imaging studies [26,27]. The two calcium imaging studies using two-photon microscopy observed extremely low calcium transient rate (0.1~0.5 events/min)[26,27]. Our present result (0.17 events/min) was in the range of previously reported values, also indicating that our method successfully extracted calcium activity at cellular resolution. We did not analyzed place-related activity of the GCs, because we did not collect sufficient number of calcium transient for reliable estimation of place field in a recording session (5 min), owing to their extremely low calcium transient rate. Much longer recording time is needed to determine place field properties of the dentate GCs.

In this work we used conventional optical components and a low-cost, general purpose CMOS camera at a cost of about 500 USD (Fig 1A and 1B and S1 Fig). This system exhibited sufficient performance for *in vivo* calcium imaging of dentate GCs (Fig 3). However, instead of the low-cost camera used in this study, high sensitivity, low noise cameras, such as cooled CMOS cameras or electron-multiplying charge coupled device (EM-CCD) cameras, enable a higher frame rate and longer recording time [11].

An important application of wide-field microscopy in neuroscience is head-mount fluorescent microscope for rodents [12]. Because of its small size (~2g), imaging optics should be simple and small-size. Therefore, in most cases, GRIN lens is used as objective lens. However, due to its low NA (<0.6) and optical aberrations, the resolution is not as good as conventional microscope objectives. To improve image quality of GRIN lens-based imaging system, compound lens system [32] or adding correction optics [33] have been proposed. The former type of GRIN lens–based, high NA (0.8) probe have been commercially available (GRINTECH GmbH, Jena, Germany). The probe is small diameter (1 mm) and offers diffraction-limited spatial resolution. Due to its small diameter, the probe can be implanted in animal’s brain. Therefore it is suitable for visualization of deep brain structures. One shortcoming of the probe is narrow field of view (~75 μm diameter)[34]. On the other hand, our method offers larger field of view (~1 mm for our present implementation). However, imaging depth is restricted by the working distance of the objective lens located outside the brain tissue. Overall, these two methods are complementary to each other.

The optical path of our imaging system (Fig 1B) is easily expandable to dual-color imaging. This modification will allow dual-color imaging of various förster resonance energy transfer (FRET) biosensors [35–37] or dual-color calcium imaging [38]. Overall, the low cost, easy-to-build high-resolution imaging system described here would facilitate research using deep brain functional imaging of behaving animals.

## Supporting information

S1 Fig Schematic diagram of the custom wide-field fluorescent microscope. Mechanical and optical components necessary to construct the microscope were shown.
(PDF)

S2 Fig XY and XZ views of a formaldehyde-fixed mouse brain expressing GCaMP6f. Eighty XY images were taken with intervals of 2 μm (Top) and a 3D tissue volume was reconstructed from the fluorescence images. XY (Bottom, left) and XZ (Bottom, right) views of the 3D tissue volume were shown.
(PDF)

S1 File Example movie of calcium imaging from dentate gyrus. An example of dF/F format data recorded from the dentate gyrus in a mouse running along a virtual track (animal id:dg4-2). The calcium imaging data was acquired at 10 fps and the movie is displayed at 30 fps. dF/F was calculated as follows; dF/F = (F(t)-F0)/F0, where F0 is the mean image obtained by averaging the entire movie, and F(t) is every frame in the movie.
(AVI)

S2 File ARRIVE guidelines. NC3Rs ARRIVE guidelines checklist.
(DOCX)
