# Supplementary figures and images for: In vivo calcium imaging from dentate granule cells with wide-field fluorescence microscopy
Source: PLoS One. 2017 Jul 12;12(7):e0180452. doi: 10.1371/journal.pone.0180452 (PMC5507494; doi:10.1371/journal.pone.0180452)

Supplementary Figure 1

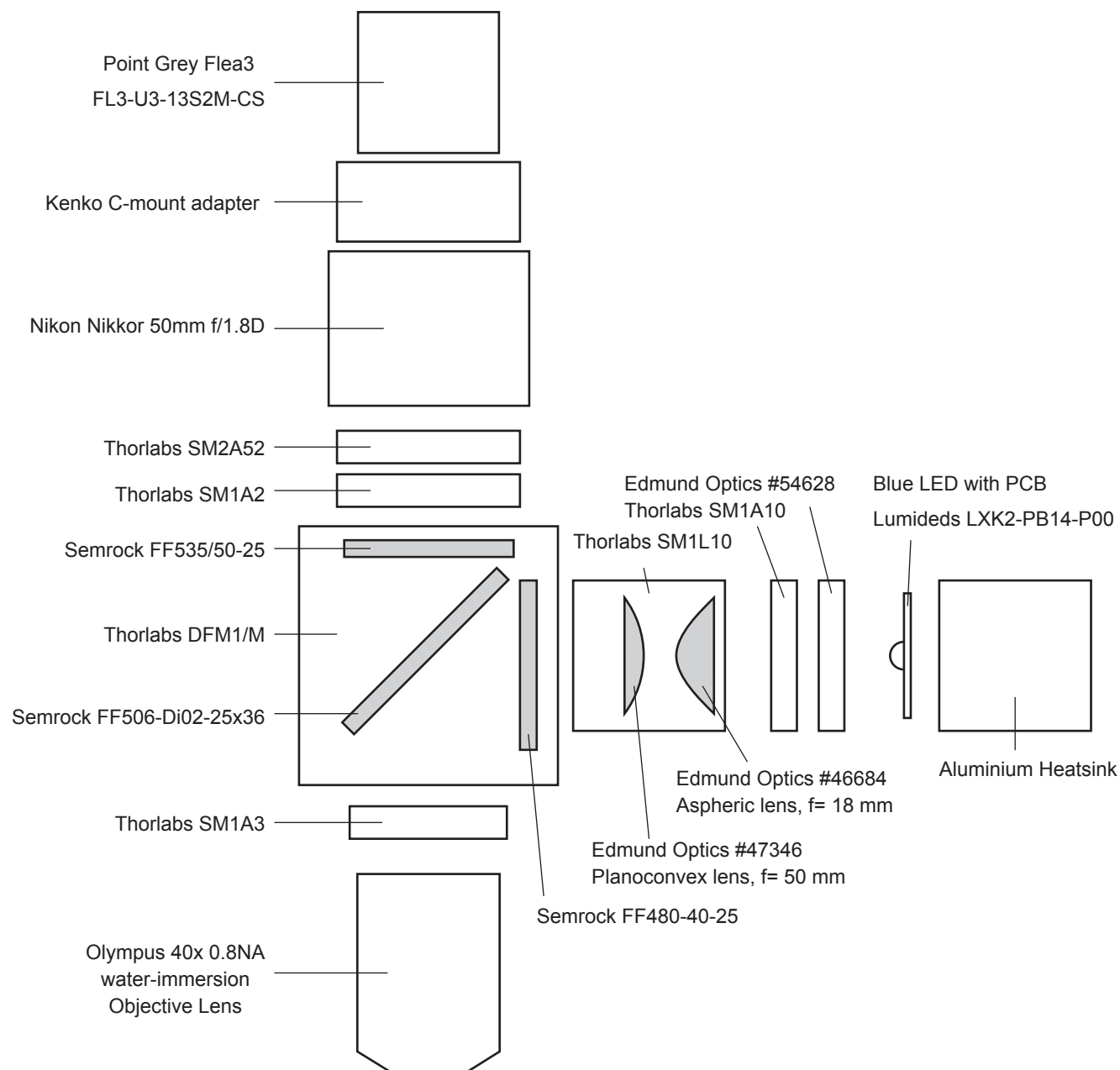

Supplement: S1 Fig — Mechanical and optical components necessary to construct the microscope were shown. (PDF) [file pone.0180452.s001.pdf]

Supplemental Figure 2

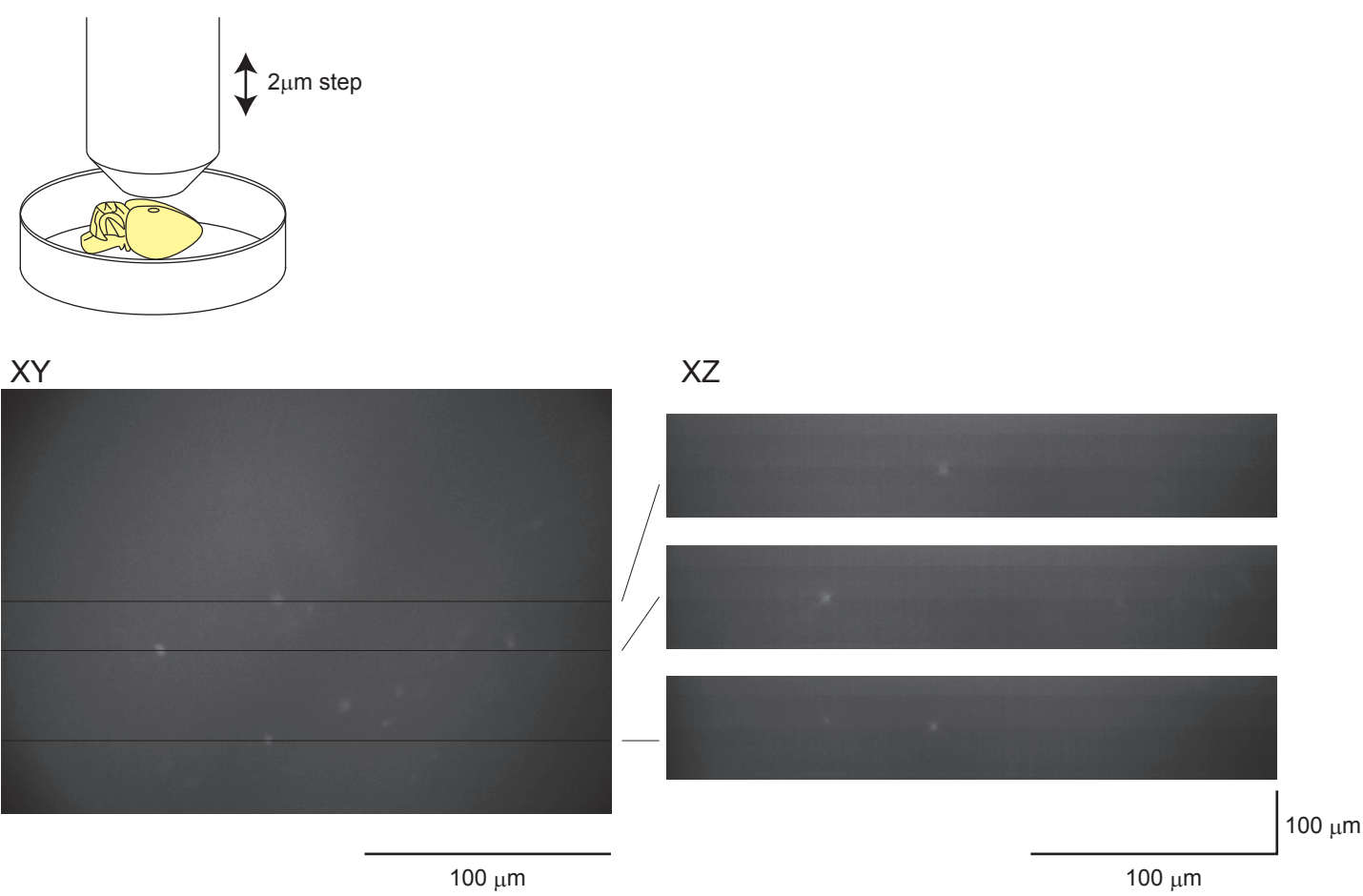

Supplement: S2 Fig — Eighty XY images were taken with intervals of 2 μm (Top) and a 3D tissue volume was reconstructed from the fluorescence images. XY (Bottom, left) and XZ (Bottom, right) views of the 3D tissue volume were shown. (PDF) [file pone.0180452.s002.pdf]
