# Supplementary material for: In vivo calcium imaging from dentate granule cells with wide-field fluorescence microscopy
Source: PLoS One. 2017 Jul 12;12(7):e0180452. doi: 10.1371/journal.pone.0180452 (PMC5507494; doi:10.1371/journal.pone.0180452)

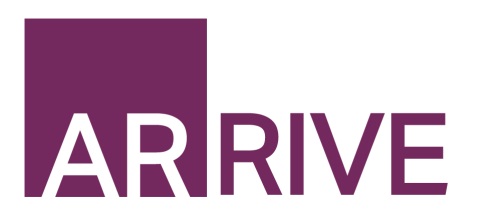


The ARRIVE Guidelines Checklist

Animal Research: Reporting In Vivo Experiments

Carol Kilkenny^1^, William J Browne^2^, Innes C Cuthill^3^, Michael Emerson^4^ and Douglas G Altman^5^

*^1^The National Centre for the Replacement, Refinement and Reduction of Animals in Research, London, UK, ^2^School of Veterinary Science, University of Bristol, Bristol, UK, ^3^School of Biological Sciences, University of Bristol, Bristol, UK, ^4^National Heart and Lung Institute, Imperial College London, UK, ^5^Centre for Statistics in Medicine, University of Oxford, Oxford, UK.*

|  | | ITEM | RECOMMENDATION | Section/ Paragraph |
| --- | --- | --- | --- | --- |
| 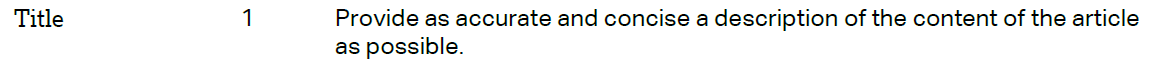 | | | Title |  |
| 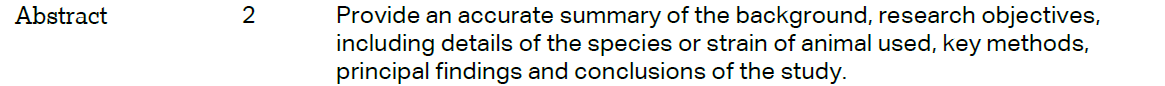 | | | Abstract |  |
| INTRODUCTION | | |  |  |
| 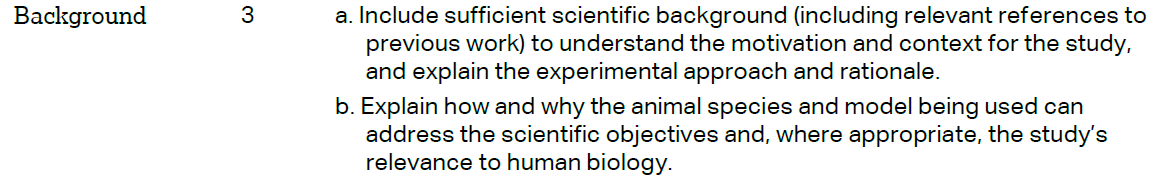 | | | Introduction, paragraph 1 |  |
| 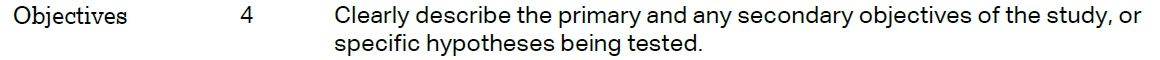 | | | Introduction, paragraph 1 |  |
| METHODS | | |  |  |
| 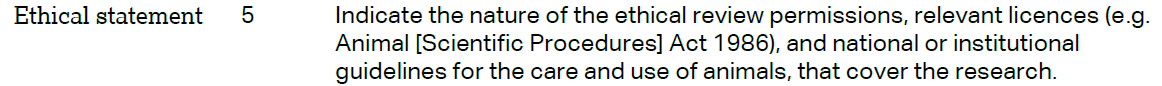 | | | Methods, paragraph 3 (Animals and surgery) |  |
| 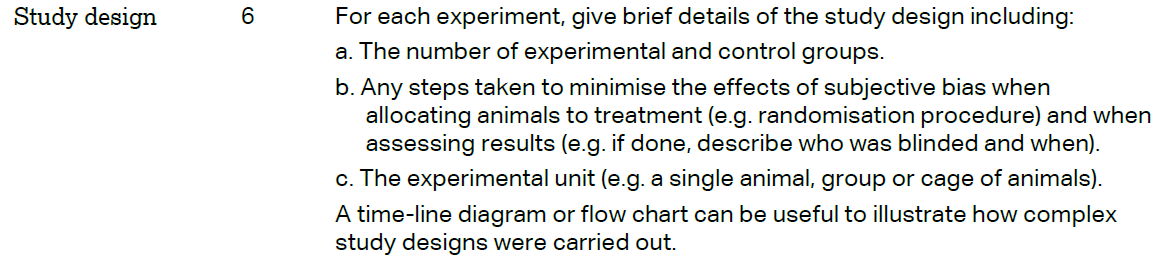 | | | Methods, paragraph 3 (Animals and surgery), 5 (Behavioral training), and Results, paragraph 2 (Activity profile of the dentate GCs) |  |
| 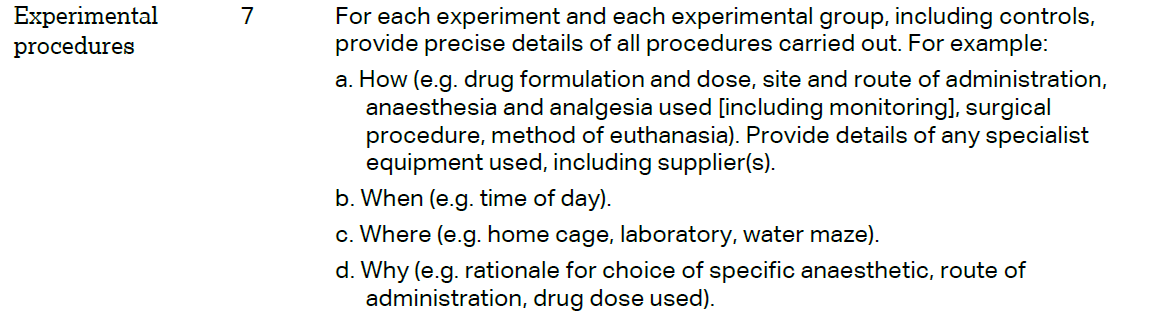 | | | Methods, paragraph 3 (Animals and surgery), 4 (VR system), 5 (Behavioral training), and Results, paragraph 1 (Wide-field fluorescence imaging of dentate GCs in awake behaving mice) |  |
| 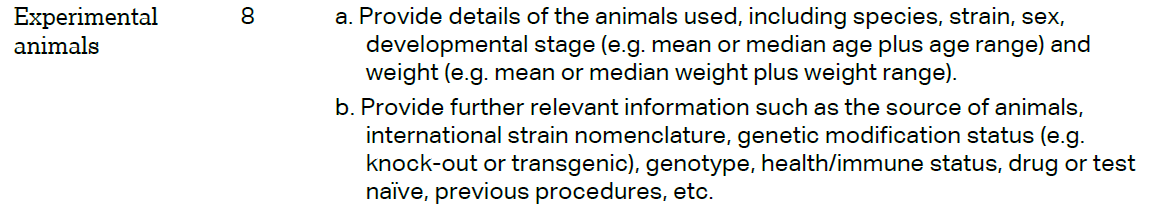 | | | Methods, paragraph 3 (Animals and surgery) |  |

The ARRIVE guidelines. Originally published in *PLoS Biology*, June 2010^1^

| 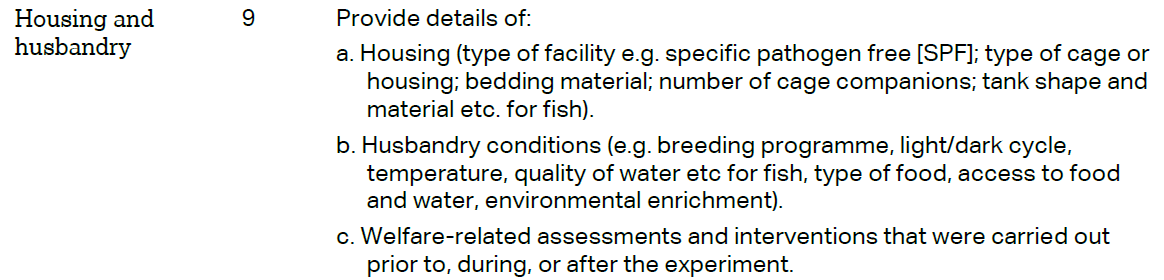 |  | |
| --- | --- | --- |
| 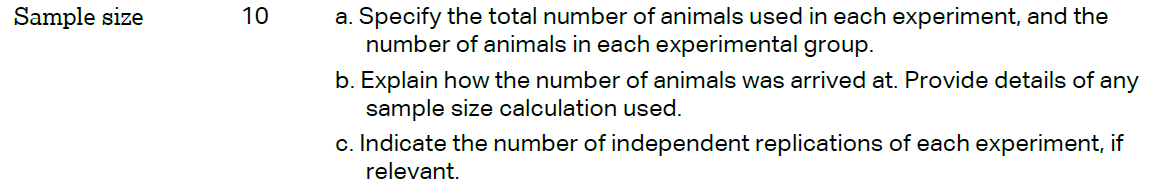 |  | |
| 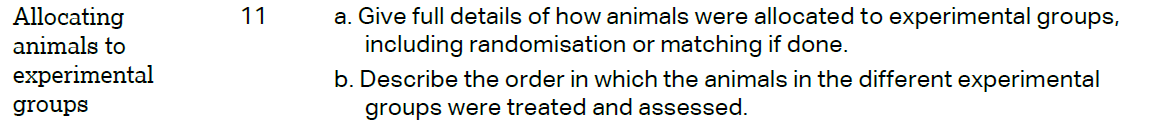 |  | |
| 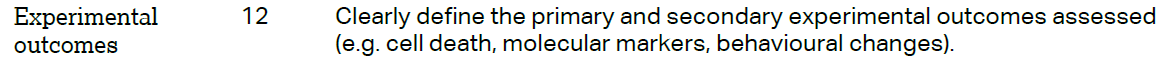 |  | |
| 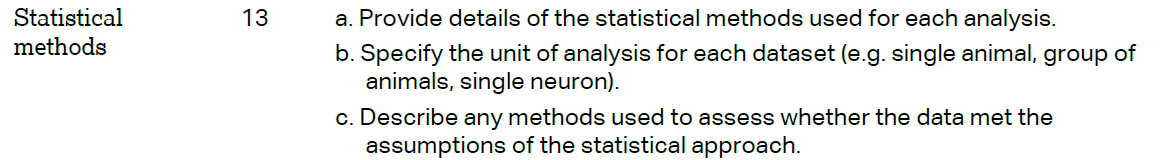 |  | |
| RESULTS |  | |
| 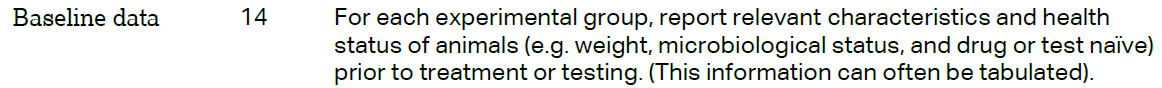 |  | |
| 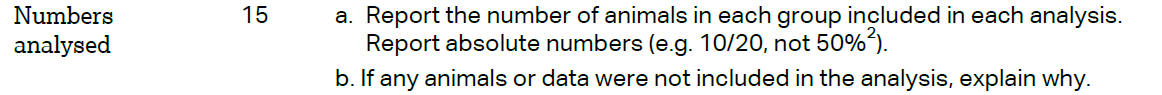 |  | |
| 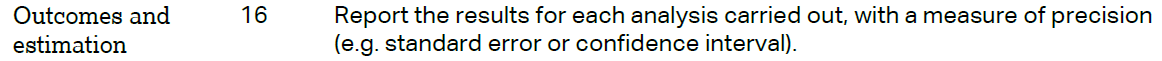 |  | |
| 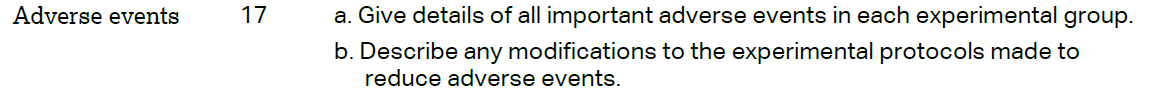 |  | |
| DISCUSSION |  | |
| 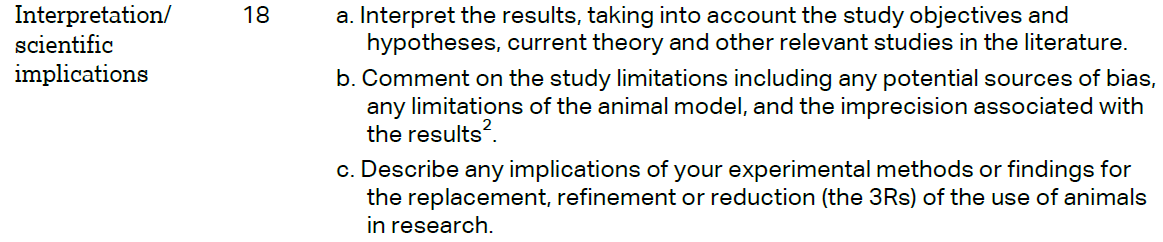 |  | |
| 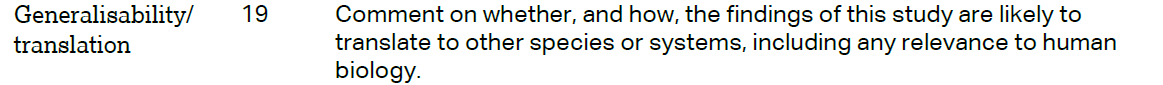 |  | |
| 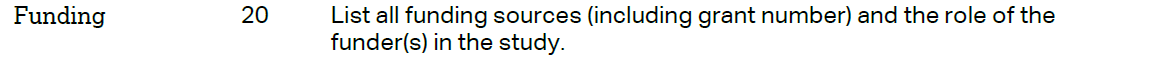 | |  |


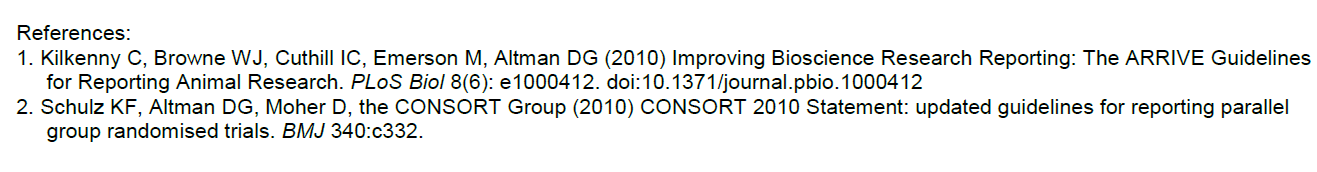

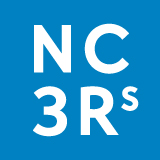

Supplement: S2 File — NC3Rs ARRIVE guidelines checklist. (DOCX) [file pone.0180452.s004.docx]
